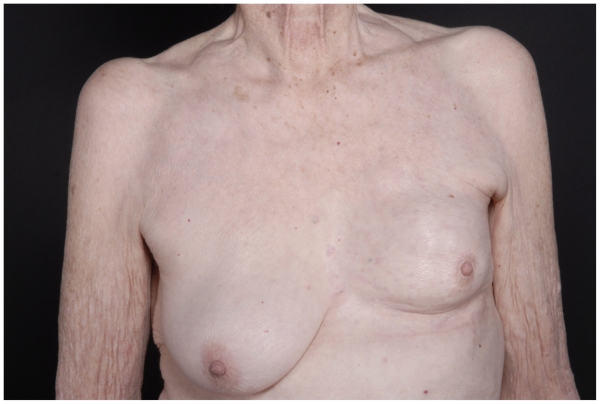# Response of Cutaneous Metastases from Breast Cancer to Capecitabine

**DOI:** 10.4137/cmo.s521

**Published:** 2008-04-18

**Authors:** Kostandinos Sideras, Katie M. Zahasky, Judith S. Kaur

**Affiliations:** Division of Medical Oncology, Mayo Clinic College of Medicine, Rochester, MN, U.S.A

## Case 1

**Figure 1:**
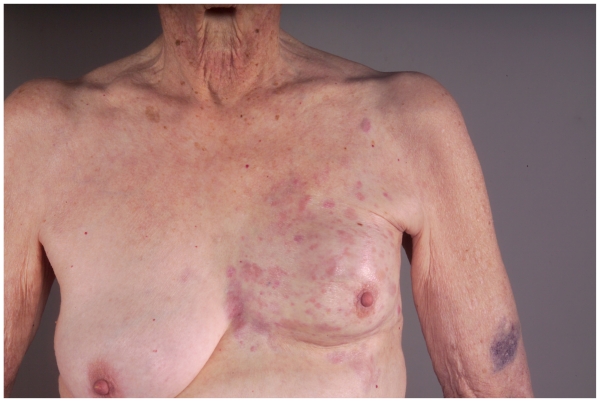


An 82 year old female with a history of ductal carcinoma in situ of the left breast in 1985, treated with excision and radiation therapy at that time, presented in July of 2003 with a shrunken and firm left breast and multiple erythematous papules on the skin. Biopsy showed an estrogen and progesterone receptor positive, invasive lobular carcinoma. Bilateral mammograms were negative as well as CT scans of the chest, abdomen and pelvis. Letrozole was initiated and the patient experienced stable disease until July of 2004 when progression was noted. Hormonal therapy was switched to Tamoxifen, and the patient initially experienced a partial response and then had stable disease until May of 2005 at which time progressive disease was noted again with multiple new nodules over the left breast and chest wall (Fig. 1). Metastatic workup was again negative. Patient was started on 850 mg/m^2^ of single agent capecitabine twice a day, on a 14 out of 28 day schedule modified for her advanced age, creatinine clearance of 18 and chronic cytopenias, but developed renal insufficiency and grade 2 diarrhea. Capecitabine was further modified to 700 mg/m^2^ on a 5 day out of 28 day cycle, which she tolerated much better. Despite dose reduction she experienced a significant response after only 2 cycles and had no progression after 10 cycles of treatment (March 2006) at which time Capecitabine was discontinued due to progressive renal insufficiency (Fig. 2). The patient experienced no progression until another 7 months when disease was found in the liver and hospice care was initiated. She expired 6 months later (June, 2007).

## Case 2

A 61 year old female was diagnosed with an estrogen and progesterone receptor positive stage II breast cancer of the right breast in 1995. She was initially treated with modified radical mastectomy, 6 cycles of adjuvant Cyclophosphamide, Doxorubicin, and Fluorouracil followed by 5 years of adjuvant Tamoxifen. In late 2003 patient was found to have biopsy proven recurrence in a right supraclavicular node and CT showed a soft tissue mass in the right axillary area. Palliative treatment with Anastrozole was initiated and the patient experienced a clinical response. In October of 2004 CT scan showed enlargement of the axillary mass. The patient was treated with 6600 cGy of radiation therapy with weekly Taxol as radiosensitizer. Her hormonal therapy was switched to Exemestane. Patient was found to have progressive disease in May of 2005 when several firm subcutaneous nodules were detected in the right axillary fold and lateral chest wall. Patient was started on capecitabine 1500 mg/m^2^ twice a day on a 14 day every 21 day schedule and had to be gradually modified to a 14 days every 5 week schedule due to cytopenias. Significant response was seen clinically after 2 cycles, a complete response was observed after 3 cycles and the patient remained without progression for 14 months (10 cycles) during which time progression was noted. She experienced grade 1 fatigue, alopecia and stomatitis as well as grade 1 hand-foot syndrome which improved with gradual modification of the regiment. After progression she was sequentially treated with single agent gemcitabine (partial response) and examestane (no response) before initiating hospice care fore the last 6 months of her life and expiring in December 2007.

## Discussion

Excluding malignant melanoma, breast cancer has the highest incidence of cutaneous metastasis compared to any other solid malignancy. Autopsy studies reported an estimated incidence of 24%.^1^ In a review on 420 patients with cutaneous metastatic disease by Lookingbill et al. 70.7% of the skin metastasis in female patients were from breast cancer.^2^

The clinical presentation varies as 80% of the lesions are papules and nodules and 11% are telangiectatic carcinoma.^3^ Other presentations include erysipeloid carcinoma, “en cuirasse carcinoma”, alopecia neoplastica, zosteriform pattern, and melanoma like pigmented lesions.^3^–^5^

In a review of 164 cases of breast cancer with cutaneous metastasis conducted by Mordenti et al., the commonest anatomic sites involved were the sites of previous mastectomy and the anterior aspect of the chest.^3^ These two locations compromised over three quarters of the cases studied. Less common sites included the axilla, back, scalp, periauricular area, supraclavicular area, face, neck, and upper and lower extremities. In the study by Lookingbill et al., there was a greater variability in the distribution of metastatic disease by anatomic location.^2^ The eyelids have also been found to be targets for metastatic breast disease (carcinomatosis blepharitis).^6^

Little is known on specific treatment of metastatic breast cancer to the skin and thus systemic treatment modalities for metastatic breast cancer in general are routinely employed. Surgery and radiation therapy are usually avoided (unless in the palliative setting) since skin metastasis is commonly an indication of more advanced disease. This is not true however when dealing with isolated locoregional recurrence, which occurs anywhere from 2.3% to 14% of women treated today for localized breast cancer, depending on the modality of treatment (mastectomy versus lumpectomy and radiation).^7^,^8^ Locoregional recurrence can occur anywhere from months to over 10 years following primary therapy, and surgical treatment can result in significant disease free survival. When locoregional recurrence involves primarily the skin, as for example the site of the surgical scar, surgical therapy (such as mastectomy if a lumpectomy was earlier performed) is standard and re-irradiation of the chest wall is not uncommon.^9^ Frequently however, the skin involvement is a direct extension of a deeper primary, and when the chest wall is involved, a full thickness chest wall excision is usually performed. Although this approach can cure a few women, there can be significant complications with direct implications in quality of life.^10^,^11^ Occasionally such extensive chest wall operations have been performed in the palliative setting in women with known metastatic disease to other organs.^12^

In general a single cutaneous lesion is associated with a better prognosis than multiple metastatic sites. In a study by Fentiman et al., 22% of patients with a single cutaneous lesion survived over 10 years.^13^ Photodynamic therapy has been employed in the treatment of cutaneous metastasis of breast cancer with complete response rates ranging from 13.5% to 40%.^14^,^15^ However, candidates for photodynamic therapy usually exhibit only small volume disease. In one study 16 skin lesions in 12 patients were treated with intralesional interferon alpha injections with 7 complete responses and 7 partial responses.^16^

Topical agents have also been tested with poor to minor results. Miltefosine, a topical cytostatic agent, was applied in 25 patients with skin metastasis and a 36% response rate was observed. However, the majority of the responses were minor.^17^ In another study topical ceramides yielded a partial response in only one of 25 patients with cutaneous breast cancer.^18^

No particular systemic treatment has been suggested specifically for cutaneous metastatic breast cancer. Capecitabine has shown a response rate ranging from 15% to 28% in the setting of metastatic breast cancer in general with complete response rates ranging from 1%–4%.^19^–^22^ In these studies, the incidence of cutaneous metastasis is either not provided,^19^,^20^ or when provided, the response of the skin lesions are not described separately (25% of 136 patients had skin lesions in the study by Reichardt et al.).^21^

Our two patients who received capecitabine showed a dramatic response with resolution of their skin lesions that lasted over a year. There is no evidence to suggest that capecitabine has particular activity against cutaneous metastasis from breast cancer. However, we believe that these two cases could potentially encourage documentation of cutaneous specific responses from capecitabine.

## Figures and Tables

**Figure 2:**